# Advancing Dynamic-Time Warp Techniques for Correcting Eye Tracking Data in Reading Source Code

**DOI:** 10.16910/jemr.17.1.4

**Published:** 2024-03-18

**Authors:** Naser Al Madi

**Affiliations:** Department of Computer Science, Colby College, USA

**Keywords:** Eye movement, Reading, Gaze, Source Code, Eye Tracking, Correction, Drift

## Abstract

**Background:** Automated eye tracking data correction algorithms such as Dynamic-Time Warp always made a trade-off between the ability to handle regressions (jumps back) and distortions (fixation drift). At the same time, eye movement in code reading is characterized by non-linearity and regressions.

**Objective:** In this paper, we present a family of hybrid algorithms that aim to handle both regressions and distortions with high accuracy.

**Method:** Through simulations with synthetic data, we replicate known eye movement phenomena to assess our algorithms against Warp algorithm as a baseline. Furthermore, we utilize two real datasets to evaluate the algorithms in correcting data from reading source code and see if the proposed algorithms generalize to correcting data from reading natural language text.

**Results:** Our results demonstrate that most proposed algorithms match or outperform baseline Warp in correcting both synthetic and real data. Also, we show the prevalence of regressions in reading source code.

**Conclusion:** Our results highlight our hybrid algorithms as an improvement to Dynamic-Time Warp in handling regressions

## Introduction

Eye tracking recordings in general are susceptible to a form of error where the detected fixation location drifts from the actual fixation location. The consistent deviations between participants’ intended fixation location and the position captured by the eye-tracker are referred to as systematic error (22). Systematic error in eye movement recording results from various difficulties (3), including low accuracy of the eye-tracker (34), calibration problems (16), free head movement (15), fixation detection algorithm (8), and changes in light conditions (11). These errors significantly increase the difficulty of processing, analyzing, and modeling eye movement data, especially in reading tasks, where eye movement is often studied at the word level to establish connections with human cognition (22). For example, a horizontal displacement of eye movement data shifts participants’ gaze position from one word to another, and a vertical displacement shifts the gaze position from one line to another (34). These errors could potentially invalidate the recorded experimental data and complicate the understanding of participants’ reading processes, even with more sophisticated eye trackers. One study by Reichle and Drieghe (28) shows how systematic errors in the position of fixations can falsely produce eye movement phenomena such as parafoveal-on-foveal effects and spillover effects in reading. Therefore, the recorded fixation data must be corrected prior to analysis. Various methods have been proposed to handle such errors associated with fixations in reading tasks, including manual and automated correction techniques.

Manual correction is a common approach to correct fixation positions (24). In the manual correction procedure, human correctors are given a visualization of the recorded participant’s fixations overlaid on the stimuli image, and they adjust the recorded fixations if necessary. Correctors are usually guided by fixation duration and position, saccade trajectories, textual context, as well as general knowledge of eye tracking and human reading behavior (12). Human correctors also have the option to discard fixations when necessary, especially when some fixations are far away from any text (12).

Although manual correction is frequently used, it is considered time-consuming (12; 22) and subjective (13). To reduce bias in manual correction, two or more human correctors often work side by side to correct eye tracking data simultaneously, with at least one corrector having prior experience in correcting fixation data in natural language reading (24). However, employing multiple human correctors increases the time and labor involved, resulting in an inefficient correction process in terms of time and effort. Therefore, automated correction techniques have been developed and are in high demand.

Over the years, many automated correction algorithms have been developed (1; 6; 7; 11; 17; 19; 21; 34; 35). Recently, Carr et al. (12) classified automated correction techniques into three categories based on their underlying concept and the information they utilize (the algorithm names come from Carr et al. (12)):

1. **Absolute positional algorithms:** Algorithms that use the absolute position of the fixation and closest line in the correction process, include Chain (30) and Attach (12). For example, the simple Attach algorithm relies on the absolute position of the fixation to attach it to the closest line, while the Chain algorithm groups fixations based on their position and attaches each group to its closest line. These methods focus on the absolute position of fixations and lines and are considered minimalist and conservative (12).
2. **Relative positional algorithms:** These algorithms focus on the relative position of fixations and lines and consider the overall number of lines in the trial in the correction process. Relative positional algorithms include Cluster (30), Regress (13), Merge (33), and Stretch (20). For example, the Regress algorithm fits n regression lines to the cloud of fixation data (n = number of lines) and classifies each fixation based on the regression line with the minimum error.
3. **Sequential algorithms:** Algorithms that assume that reading is sequential from top to bottom and from left to right in languages like English. These algorithms rely primarily on the order of fixations in matching fixations to lines. Sequential algorithms include Segment (1) and Warp (12).

Warp is one of the most successful algorithms in correcting eye tracking data in reading tasks, and it relies on Dynamic Time Warping (DTW) (29) to align fixations to text lines. The algorithm assumes that readers will read the text sequentially from left to right and from top to bottom, matching fixations to word centers in a way that minimizes the overall Euclidean distance between fixations and word centers. Warp and sequential methods are invariant to distortions, such as noise, because they focus on the order of fixations in addition to their positions. The main limitation of Warp and sequential algorithms is regressions, where the eyes move back to previous words or lines in violation of the sequential reading assumption. While the prevalence and magnitude of regressions can vary in natural language reading, regressions can be common in reading source code, where nonlinear reading is the main characteristic (9).

While Warp appears to outperform almost all correction algorithms, one of the main findings of Carr et al. (12) is that no single algorithm is suitable for all distortions and conditions. Algorithms that perform well with regressions tend to underperform in other situations, and algorithms that perform well with distortions tend to underperform when regressions are present. Algorithms have to make a trade-off between how well they handle distortions and regressions.

This poses a significant challenge for the automatic correction of eye tracking data in reading source code, since reading source code is characterized by regressions. Despite sharing similarities with natural text, such as the use of Latin letters and Arabic digits, source code reading differs fundamentally in purpose, syntax, semantics, and viewing strategy from reading natural language text (9; 10; 18; 31). Empirical studies on eye movement during reading reveal distinctions between natural text and source code. One major distinction is that natural text is typically read in a linear fashion (from left to right and top to bottom in English), while source code reading is less linear and characterized by regressions (jumps back). This unique reading strategy tends to increase with programming experience and resembles code tracing rather than following the sequential order of code (5; 9; 25).

In the study of human-oriented Software Engineering, the use of eye-tracking has gained traction with hundreds of studies conducted since 1990, offering valuable insights into the attention and cognitive processes of programmers (23; 32). Therefore, presenting automated algorithms for correcting eye tracking data in reading source code can be very beneficial for eye movement in programming research. In this paper, we propose a family of hybrid algorithms, which aim to enhance the performance of Warp (DTW) in correcting eye tracking data in reading source code. The proposed algorithms combine Warp with other algorithms to optimize the correction performance across trials with and without regressions.

We assess the proposed algorithms with synthetic data and two real datasets, and we compare their performance to Warp. We present a realistic synthetic data that includes typical reading phenomena such as word skipping, and we utilize real eye tracking data from reading source code (4), and examine the generalizability of the algorithms in a natural language dataset. Additionally, we provide a detailed analysis of the runtime of each algorithm, along with recommendations for researchers on how to design experiments and choose automated algorithms to correct data and maximize correction quality based on our findings. Also, we make our code and data publicly available through a replication package.

## Proposed Approach

**Figure 1:**
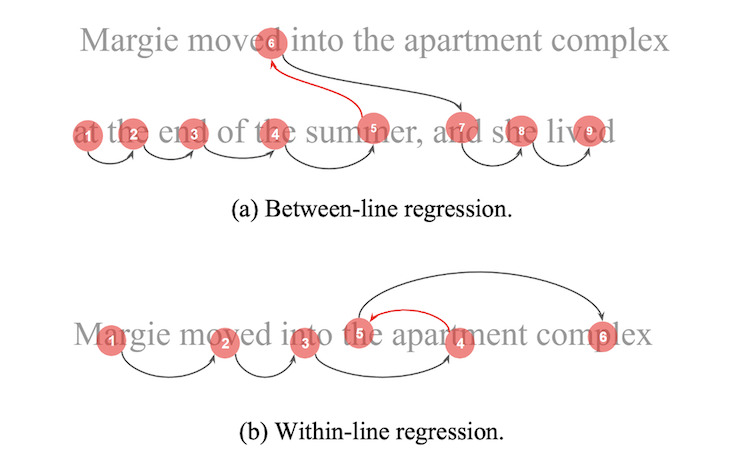
Illustration between-line and within-line regressions, where fixation order is represented by a number.

One of the main findings of Carr et al. (12) is that automated algorithms have to make a trade-off between excelling in correcting data either with regressions or with distortions, none of the algorithms reviewed performed well in both conditions. Despite the excellent performance of Warp in almost all forms of distortion, the algorithm underperforms in trials containing between-line regressions, as regressions violate the sequential reading assumption made by the algorithm, as illustrated in Figure 1. On the other hand, algorithms like Chain and Regress (13) are indifferent to regressions, but they underperform in correcting trials with typical drift distortions.

The hybrid algorithms we propose aim to advance Warp by allowing it to perform well in correcting trials with between-line regressions and distortions. The main concept behind our hybrid algorithms is detecting regressions and splitting regressions from the rest of the data, the data without regressions is corrected with Warp, then the regression is added to the correction and the combination is corrected by one of the algorithms that excel in correcting regressions.

**Figure 2:**
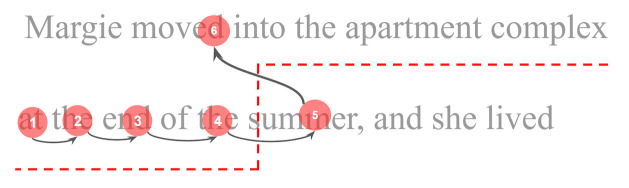
Illustration of the two areas defined by the regression detection and splitting function.

The main component that hybrid algorithms rely on is the detection and splitting of regressions from the rest of the data. The detection of regressions is based on defining two areas while progressing through fixations, as illustrated in Figure 2. For fixation number 5 in the illustration, if the next fixation is within the area above the red line, then that is considered the beginning of a regression. All following fixations are also considered part of the regression, until a fixation falls below the red line defined for fixation number 5 which preceded the regression. This regression detection technique works even if fixations are not aligned with lines of text, since the red line is defined in relation to the position of fixations, not the text. The red line takes into consideration the height of each line, which is calculated automatically from the image of the text stimulus. The red line combines within-line and between-line regressions, within-line regression is detected by equation 1 and between-line regression is detected by equation 2:


(1) x<last_x−line_height/2andy≤last_y+line_height/2

(2) y<last_y−line_height/2

Where x and y are the coordinates of the current fixation, last x and last y are the coordinates of the previous fixation, and line height is the line height. In the case of variable line height due to vertical spacing in source code, average line height is taken instead. If either of the two equations evaluates to true, then the beginning of a regression is marked and all fixations that lay above the red regression line are marked as part of the regression. The splitting of regressions from the rest of the fixations allows for using any combination of algorithms to correct the trial. After splitting regressions from the rest of the fixations, we end up with two sets of fixations, a sequential non-regressive set of fixations and a set of regressive fixations. Warp showed excellent performance in correcting distortions other than between-line regressions (12), so we chose Warp for correcting the non-regression set of fixations. After that, the regressive set of fixations is combined with corrected non-regressive set and the combination is passed to an algorithm that performs well with regressions. Combining the corrected non-regressive set and the regressive set of fixations gives the regressive algorithm more accurate data to perform its correction. We test four algorithms that perform well with regressions: Attach (12), Regress (13), Chain (30), and Stretch (20). We name the resulting combinations Hybrid(warp+attach), Hybrid(warp+regress), Hybrid(warp+chain), and Hybrid(warp+stretch) respectively.

The simple Attach algorithm relies on the absolute position of the fixation to attach it to the closest line (12), while the Chain algorithm groups fixations based on their position and attaches each group to its closest line (30). Regress is a more sophisticated algorithm that tries to fit fixations to regression lines, where each regression line is associated with a line of text (13). Cluster utilizes k-means to group fixations from each line to an associated cluster (30). Then fixations from each cluster are assigned a line from the text. On the other hand, Stretch was created for correcting source code eye movement data, and it attempts to find an offset and a scaling factor that aligns fixations with word centers (20). We use the simplified version of this algorithm that relies only on y-offset instead of x- and y-offsets.

**Figure 3:**
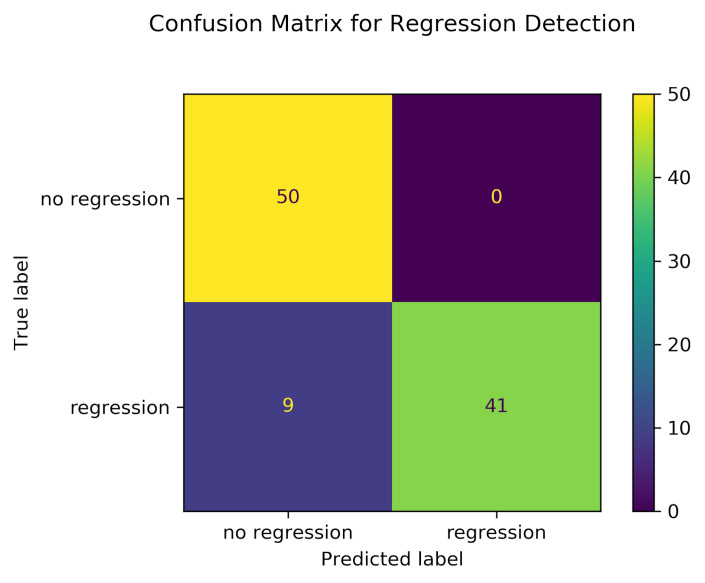
Confusion matrix for regression classification. True Negative (top-left quadrant), False Positive (top-right quadrant), False Negative (bottom-left quadrant), and True Positive (bottom-right quadrant).

To validate the regression classifier we describe above, we conduct a simulation where 100 synthetic reading trials are created, 50 of which contain exactly one regression and 50 trials contain no regressions. The regression classifier is used to classify trials as containing a regression or not. The resulting confusion matrix in Figure 3 shows True Negative (top-left quadrant), False Positive (top-right quadrant), False Negative (bottom-left quadrant), and True Positive (bottom-right quadrant). The classifier accuracy is 91%, precision is 100%, recall is 82%, F1-score is 90%.

The synthetic trials generated in the classifier validation include variations in fixation landing position, skipping, and small noise in both x and y coordinates, yet they do not include any distortions like shift, slope, or offset. Therefore, the performance of the regression classifier is expected to degrade in the presence of these distortions. We will elaborate more on which distortions affect regression detection and which do not in the next section.

## Synthetic Data Simulation

In this section, we test our proposed algorithms on synthetic data, and we compare the performance of our algorithms to Warp. This makes the synthetic data simulation an external conceptual replication of the synthetic data simulation of Carr et al. (12) with differences in algorithms, the synthetic data used, and one additional type of distortion (offset). We use the implementation of Warp, Attach, Regress, Chain, and Stretch provided by (12) to eliminate any implementation variations that might affect the results and our comparison.

**Figure 4:**
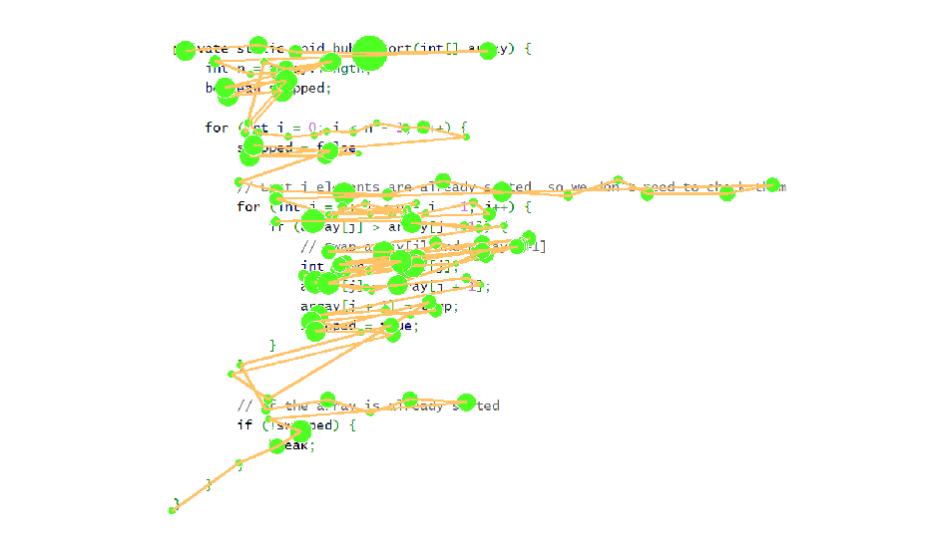
Illustration of realistic synthetic data including normal reading behavior like word skipping. The size of the fixation is proportional to its duration.

### Methods

We generate realistic synthetic data that includes normal reading behavior like word skipping and regressions, as illustrated in Figure 4. The code of the synthetic data is a Java function that performs Bubble Sort, and it includes typical variable names and comments. Synthetic fixations are positioned within 15px of the optimal viewing position of each word, which tends to be slightly to the left of the center of the word in English (26, 27). In the synthetic data, short words have 75% probability of being skipped, and we use 100 trials similar to the one illustrated in Figure 4 as our synthetic dataset.

In addition to the two types of regressions (within-line and between-line) illustrated in Figure 1, we conduct simulations with four types of distortions found in eye movement data in reading tasks. Noise, Slope, and Shift are replications of the distortions presented by Carr et al. (12), and Offset is a type of distortion that we add. Each simulation starts with a trial similar to the one in Figure 4, then distortion is introduced with a certain magnitude, after that the algorithms attempt to correct the distorted trial. The correction of each algorithm is compared with the original trial before distortion was introduced to measure the correction accuracy of the algorithm. Distortion is introduced in gradations to show how the algorithms perform under different intensities of distortion. Each form of distortion is introduced using a generator function, we elaborate on the details of each generator next.

**Figure 5:**
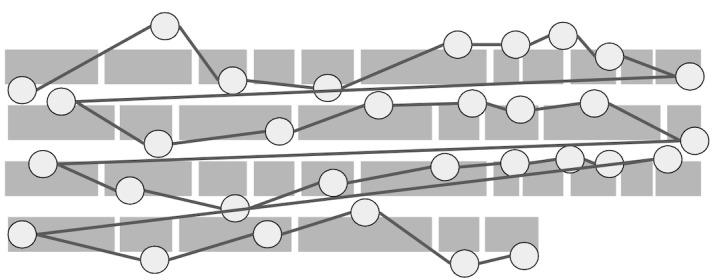
Illustration of noise distortion.

The noise generator has one parameter, y-coordinate noise magnitude. For each fixation, a random number is generated using Gaussian distribution where the mean is zero and the standard deviation is equal to noise magnitude. The generated number is then added to the y-coordinate of the fixation. The noise magnitude parameter was given values ranging between zero (no noise) and 20 (maximum noise). This results in a distortion where some fixation positions are changed, as illustrated in Figure 5.

**Figure 6:**
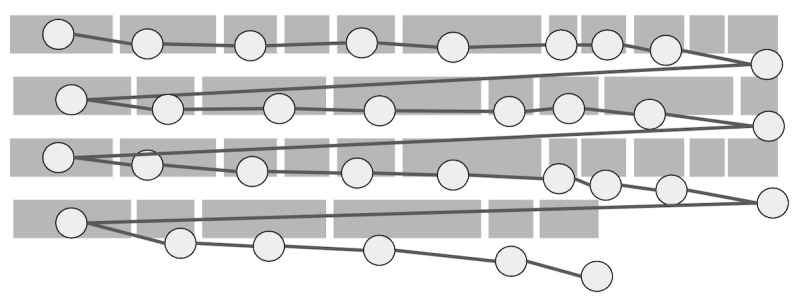
Illustration of slope distortion.

The slope generator has one parameter which controls how much fixations are moved from their original position. The further the fixation is on the x-access from the leftmost fixation, the greater the distortion introduced to the y-coordinate of the fixation, as illustrated in Figure 6. This results in a gradual distortion in the position of fixations as they approach one side of the screen.

**Figure 7:**
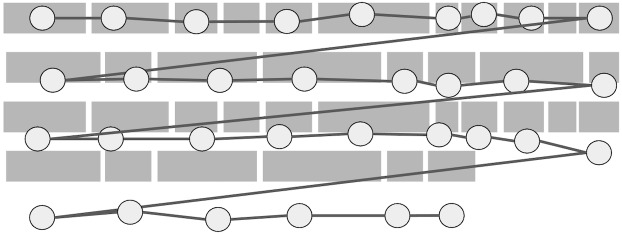
Illustration of shift distortion.

The shift distortion is similar to slope with distortion that gets progressively stronger from the top to the bottom of the screen. The shift generator has one parameter that controls the magnitude of distortion introduced to the y-access of fixations. The distortion introduced is also proportional to the distance of the fixation from the first line in the trial, as illustrated in Figure 7. This results in gradual distortion as fixations move away from the first line in the trial.

**Figure 8:**
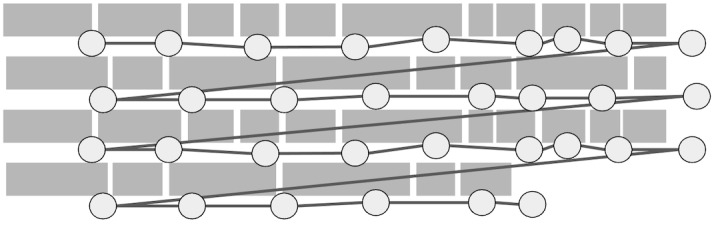
Illustration of offset distortion.

The offset generator has one parameter that controls the magnitude of distortion introduced to all fixation on the y-access. Offset distortion affects all fixation to the same magnitude by moving them on the y-access, as illustrated in Figure 8. This is a new category of distortion that was not examined by Carr et al. (12). The regression generators have two parameters, first a list of Areas-Of-Interest (AOIs) that surround each word, and the second is the probability of regression desired. Using a random number generator, the regression probability is used to randomly chose a previous word to regress to. Within-line regression generator guarantees that the regression word is on the same line as the current fixation, while between-line regression generator guarantees that the regression is on a previous line.

### Correction Results

In this section, we present the results of running Warp and along with the five proposed hybrid algorithms. For each type of distortion and regression, we run 100 simulations with 11 gradations of distortion resulting in 6600 simulations. The goal of this simulation is to see if the proposed algorithms can handle distortions and regressions with accuracy overcoming the main limitation of Warp. Ideally, the proposed algorithms match Warp’s performance in handling distortions and outperform it in handling between-line regressions.

**Figure 9:**
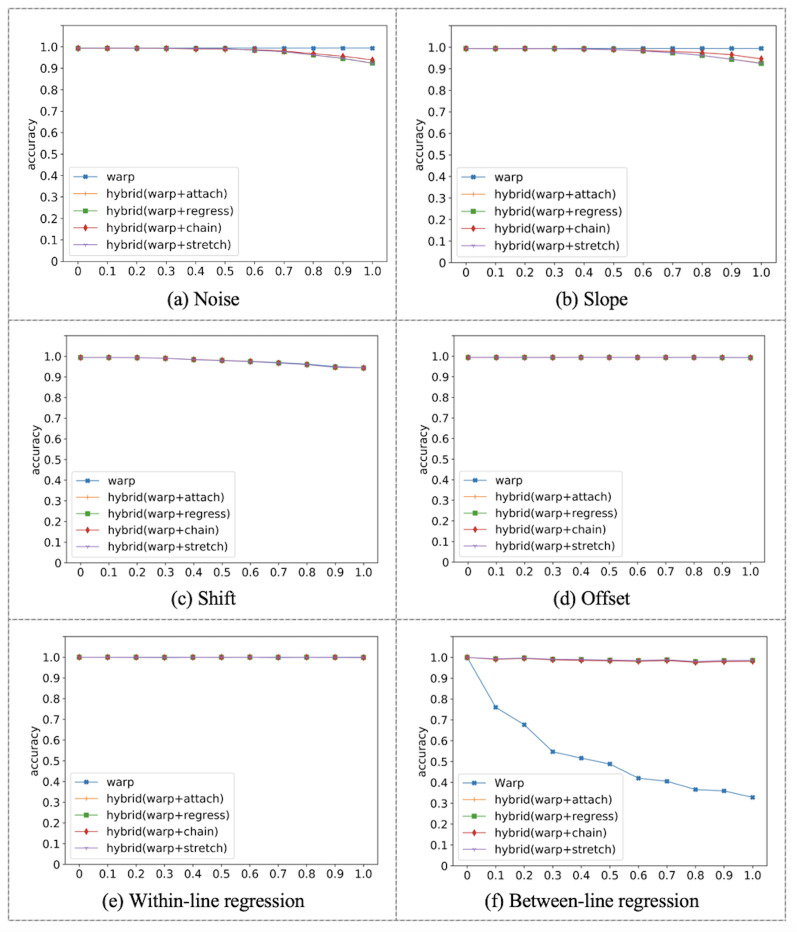
Algorithms mean accuracy in correcting distortions and regressions.

The results from our synthetic data simulations are reported in Figure 9. The five algorithms appear mostly invariant to noise distortion, as seen in Figure 9a. Warp was completely invariant to noise, while the hybrid algorithms were susceptible to error at high magnitudes of noise distortion. More specifically, when the noise was larger than line height, hybrid algorithms mistakenly classify that as a regression to a previous line, while in fact the fixation was moved above the line due to noise. The same behavior can be seen with slope distortion, where return sweeps could be incorrectly classified as regressions if the slope is larger than line height, as seen in Figure 9b.

With Shift, hybrid algorithms match Warp, and all algorithms appear to be susceptible to small amount of error when shift distortion is large, as seen in Figure 7. This small error is somewhat unique to source code, as most programs end with several curly brackets to end the scope of a block of code, as seen at the bottom of Figure 4. Since some of these curly brackets are often skipped, Warp and hybrid algorithms made errors in assigning fixations to the correct curly bracket, when shift distortion was large. In addition, all algorithms were invariant to offset distortion and within-line regressions as seen in Figures 9d and 9e respectively.

The main goal behind hybrid algorithms is to improve the performance of Warp in handling between-line regressions. Between-line regression is one of the major challenges for sequential correction algorithms, and Warp had an average accuracy of 53.3%, as seen in Figure 9f. All other hybrid algorithms had an average score between 98.6 and 98.9%. Hybrid algorithms appear unaffected by between-line regressions at all magnitudes. Overall, Warp was outperformed by all proposed algorithms.

**Figure 10:**
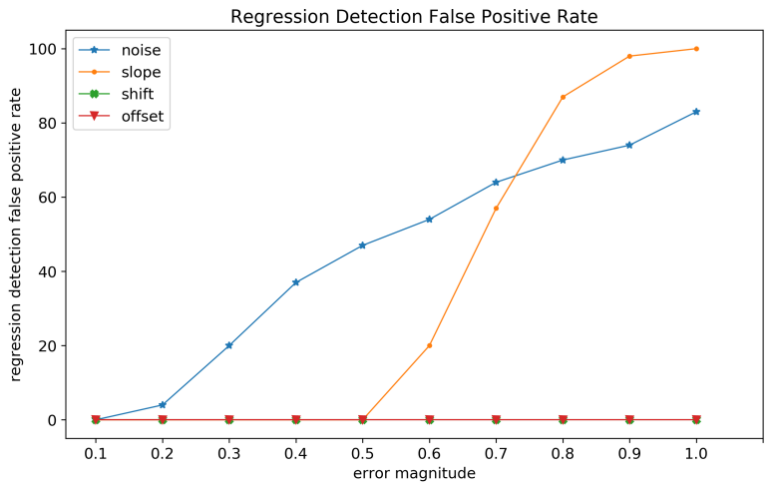
Regression detection false positive rate in relation to slope, shift, and offset distortion magnitude.

As mentioned previously, the regression detection mechanism we use is influenced by some types of distortion as it relies on fixation positions in detecting regressions. To find the types of distortion that affect regression detection we conduct a simulation with 100 trials without regressions, and introduce noise, slope, shift, and offset distortions gradually to see how each type of error affects regression detection.

Figure 10 shows the false positive rate at each gradation of error. Offset and shift distortions appear to have no effect on regression detection across all magnitudes of distortion, as they move all fixations without distorting the alignment of fixations on the same line. On the other hand, noise and slope appear to influence regression detection as they affect the positions of some fixations more than others, causing a false positive regression detection. This means that with higher distortion magnitude more trials are mislabeled as containing regressions when they do not. This has an effect on our heuristic for hybrid algorithms. At the same time, mislabeled regressions are corrected by attach/chain/regress/stretch, therefore the mislabeling at high magnitudes of error does not invalidate the regression detection heuristic as results show that it still matches or outperforms Warp. Nonetheless, this result motivates a better regression detection heuristic as a future direction.

**Figure 11:**
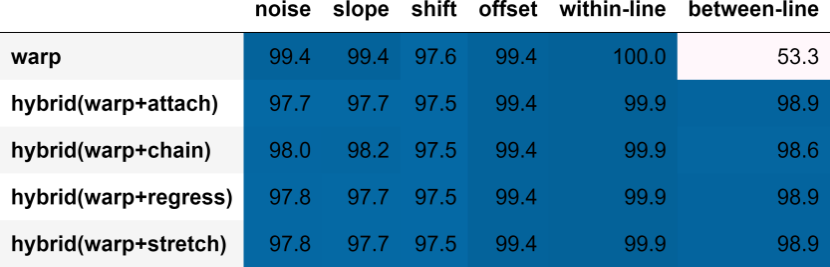
Algorithms mean accuracy in correcting trials containing distortions (accuracy %).

One of the main results from Carr et al. (12) that motivated this paper is that some algorithms handle regressions well and others do well with distortions, but a single algorithm was not able to do both. Figure 11 shows a comparison of the mean accuracy in correcting trials containing distortions and regressions. Warp performs well with distortions and within-line regression, but it underperforms in correcting between-line regressions. On the other hand, hybrid algorithms appear to do well in correcting distortions and handling both types of regressions.

### Runtime Analysis

In this section we focus on the average runtime per-trial for each of the 9 algorithms that we examine. The mean duration per trial information reported in this section are measured during the same simulations presented in the previous section, therefore we compare runtimes under each type of distortion and regression, since runtimes are expected to vary under different simulation conditions. The runtimes were calculated on a Windows 10 (version 22H2) computer with an Intel Core i7-9700k CPU at 3.60GHz and 32GB of main memory.

**Figure 12:**
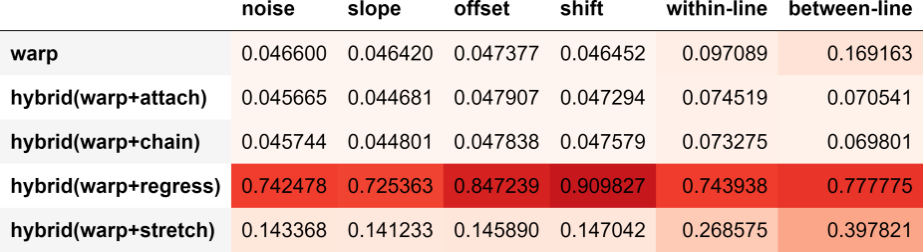
Algorithms mean runtime in correcting trials containing distortions (time in seconds).

Algorithms runtimes appear consistent under different simulation conditions of distortion and regression, as seen in Figure 12. Simulations with regression have more fixations, which might explain why within-line and between-line regressions take more time with all algorithms. In regard to our proposed algorithms, hybrid algorithms that use Regress and stretch take more time per trial compared to Warp. At the same time, hybrid algorithms that use Attach and Chain match or take less time than Warp, especially in Between-line regressions. Attach and Chain are simple algorithms that give hybrid Attach and hybrid Chain an advantage in runtime when regressions are present. When the fixations are split into two parts in hybrid algorithms, Warp works with fewer fixations and Attach/Chain are significantly faster than Warp in correcting the combined fixations.

### Summary

In terms of algorithm accuracy on synthetic data, the results suggest that hybrid algorithms match or outperform Warp in correcting distortion and handling regressions, overcoming one of the main obstacles reported by Carr et al. (12). This is a significant improvement for correcting eye tracking data in reading source code, considering how common regressions are in reading source code.

Regarding runtime, hybrid algorithms that use Attach or Chain are the fastest algorithms, and both had a very good performance in correction accuracy as well. In addition, while most algorithms show significant increase in runtime when regressions are present, hybrid Attach and Chain algorithms show marginal increase in runtime. Generally, it appears that the number of fixations is more influential on runtime than distortion type, as trials with regressions have more fixations. The proposed hybrid algorithms that use Regress or Stretch take significantly longer than warp. This means that some proposed algorithms (hybrid Attach and Chain) outperform Warp and have a shorter runtime when regressions are present. These promising results motivate testing the proposed algorithms with real data and comparing their performance to base-line Warp.

## Performance on Real Data

**Figure 13:**
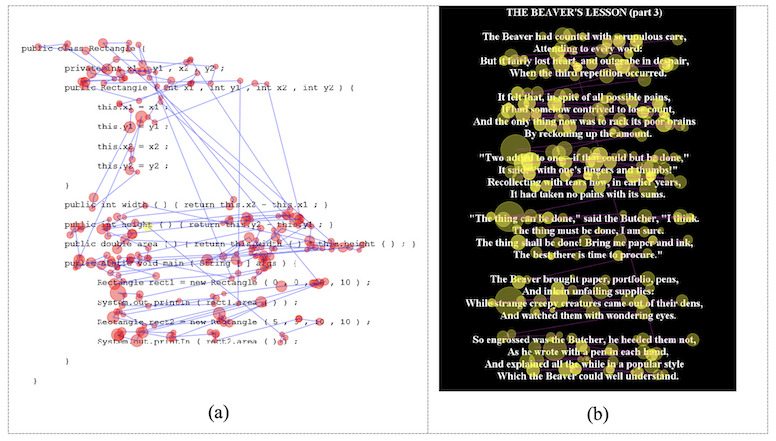
Samples from: (a) EMIP dataset. (b) GazeBase dataset.

In this section, we compare the performance of our proposed algorithms to Warp on real eye tracking data. Starting with our main objective, we utilize a source code reading dataset named the Eye Movement In Programming dataset (EMIP) (4). Then we assess the generalizability and usefulness of the proposed algorithms in correcting data from natural language reading. One dataset contains many lines of text and many regressions (GazeBase) (14). Figure 13 shows a sample eye tracking recording from each trial.

Each dataset has a unique characteristic that makes it insightful in the context of correcting eye tracking data. Using multiple datasets with different conditions provides a more accurate assessment of the correction algorithms we compare. Also, in addition to measuring and comparing algorithms in terms of accuracy, one central question that we hope to answer is how often do real eye tracking trials contain regressions? And what type of regression is prevalent? Answering this question is important considering the performance trade-off previous algorithms make between distortion and regression.

### Methods

We utilize two datasets with heterogeneous reading tasks to gain insights on the performance of our proposed algorithms in reading source code and in correcting eye tracking data in general.

First, we assess our algorithms on eye tracking data in reading source code. The dataset consists of 47 trials of reading Java code from the Eye Movement In Programming dataset (EMIP) (4). The EMIP dataset was an international and multi-institutional effort that involved eleven research teams across eight countries on four continents (2). The trials include multi-line Java source code in medium sized font, and the eye tracker used is an SMI Red 250 eye tracker with 250 samples per second. The dataset offers a unique insight on correcting eye tracking data in reading source code, which is characterized by non-linear reading and significant regressions and progressions (jumps forward) (9).

To assess the generalizability and usefulness of the proposed algorithms on natural language text, one additional dataset is used. The second dataset we use consists of 24 text trials from GazeBase (14). The trials include a dense multi-line poem with small font and variable spacing between lines. This creates a challenging set of conditions for eye tracking data correction algorithms. The eye tracker used is a high-frequency EyeLink 1000. In addition, the large number of lines in each trial increases the chances of between-line regressions.

To measure the accuracy of the algorithms in correcting data from the real datasets, a manually corrected golden set of the same data is needed. Therefore, we rely on eight human correctors to manually correct the GazeBase, and EMIP datasets as ground truth. Each dataset was corrected by two people separately, then a software tool was used by a third person to scan and merge the data. If the two correctors disagree, the third corrector is presented with a visualization of both corrections to make the final decision in accepting one.

To answer our question on the presence and prevalent type of regression in real eye tracking data, we used the human-corrected goldenset to count regressions. This is important considering that the regression detection heuristic is influenced by some types of distortion (noise and slope).

### Correction Results

In this section we compare the accuracy of the proposed algorithms to Warp in correcting the three real datasets. Accuracy is measured as the percentage of fixations that agree with the golden set by being positioned on the same line.

**Figure 14:**
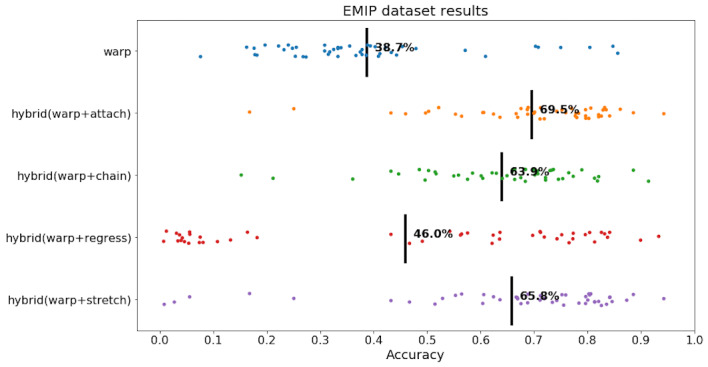
Algorithms mean accuracy in correcting trials from EMIP dataset. Black line is mean, and each trial is a dot.

The EMIP dataset is characterized by Java source code, multi-line reading, medium-frequency eye tracker, and non-linear reading pattern. Counting regression and their types in the corrected goldenset, we found that 100% of trials contained at least one type of regression, all examined trials had within-line regression and all examined trials had between-line regression.

As seen in Figure 14, Warp had an accuracy of 38.7%, and it was outperformed by all hybrid algorithms. The best algorithm was hybrid(warp+attach) with an average accuracy of 69.5%. Overall, hybrid algorithms increased the performance of Warp by 7.3 to 30.8 percentage points, which is substantial. In terms of variance in accuracy of individual trials, it appears that hybrid(warp+attach) and hybrid(warp+chain) were consist around the mean, while hybrid Regress and Stretch were more dispersed. These results suggest that hybrid algorithms like hybrid(warp+attach), hybrid(warp+chain) are substantially more successful in correcting eye tracking data with regressions compared to Warp.

The GazeBase dataset is characterized by dense multi-line text with variable line spacing, small font size, and high-frequency eye tracker. Counting regressions and their types in the corrected goldenset, we found that 100% of trials include at least one type of regression, all include within-line regressions and 95% of trials contain between-line regressions. This makes this dataset suitable for assessing the generalizability of the proposed algorithms in correcting eye tracking data in reading natural language text.

**Figure 15:**
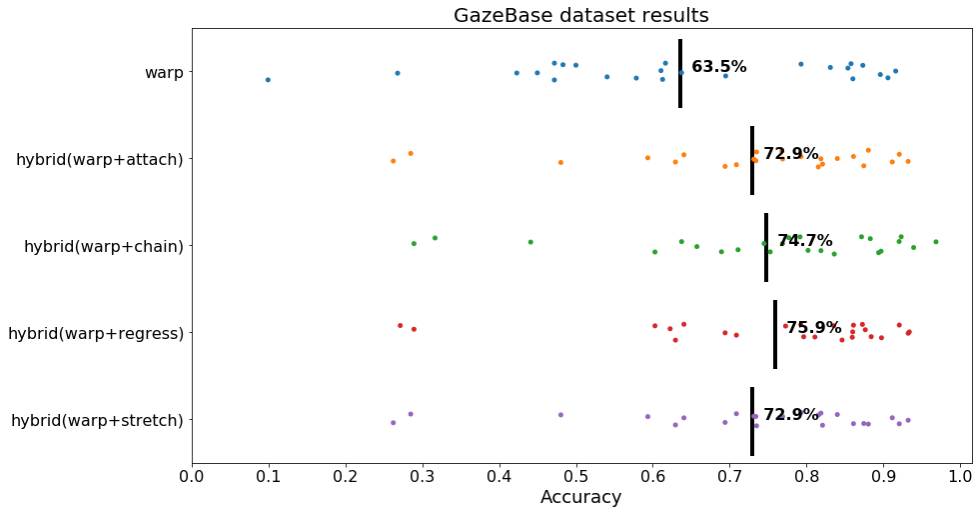
Algorithms mean accuracy in correcting trials from GazeBase dataset. Black line is mean, and each trial is a dot.

As seen in Figure 15, Warp had 63.5% accuracy. all other hybrid algorithms outperformed Warp, and the most successful was hybrid(warp+regress). All proposed hybrid algorithms outperformed Warp by 9.4 to 12.4 percentage points. We will elaborate more in the discussion section on a possible explanation for why hybrid Attach and Chain algorithms excel with source code and hybrid Regress excels with natural language reading.

### Summary

Our real data results suggest that the advantage offered by hybrid algorithms is proportional to the prevalence of between-line regressions in the data. At the same time, none of the algorithms had an accuracy above 75.9% in correcting either dataset. Overall, our proposed hybrid algorithms matched or outperformed Warp, sometimes by 30.8 percentage points.

Regarding regression rates, the results suggest that regressions are very common in real eye tracking data, especially in reading source code. Our results show that within-line regressions are more prevalent than between-line regressions, nonetheless both types are common and their presence varies possibly depending on reading “type” and task objective.

In regard to the correction accuracy in relation to the characteristics of each dataset, it appears that trials with a large font are easier to correct than smaller font, and fewer lines of text are easier to correct than many lines. The most difficult data to correct was the EMIP dataset, which is characterized by non-linear reading consisting of many regressions and progressions as typical in reading source code.

## Discussion

Considering the results from our simulations, most of our proposed algorithms appear to match or offer an improvement over Warp. With real data, our proposed hybrid algorithms matched or outperformed Warp, sometimes by 30.8 percentage points. Nonetheless, reading source code is substantially different from reading natural language text, and hence different algorithms are needed that take into account characteristics such as non-linearity. This makes correction somewhat more difficult, evident by the low accuracy scores by all algorithms when correcting eye movement over source code compared to correcting eye movement over natural language text.

In regard to synthetic data, the proposed algorithms matched or outperformed Warp in correcting data with distortions and regressions. The only exceptions are at high magnitudes of Noise or Slope distortions, where hybrid algorithms made a few mistakes by incorrectly detecting a regression when there was no regression. Nonetheless, the overall performance was still higher than 97%. Another interesting observation from synthetic data is that all algorithms, including Warp, made small correction mistakes at high magnitudes of Shift distortion. Looking at the data, it appears that these mistakes are unique to source code data, where a several lines of curly brackets are common at the end of a block of code. Some of the curly brackets were skipped due to being short, and all algorithms made mistakes in determining which curly bracket was skipped, resulting in a small percentage of errors.

In regard to real data, hybrid(warp+attach) was the best performing algorithm in correcting source code reading data. At the same time, hybrid(warp+regress) was the best performing algorithm in correcting natural language data from GazeBase. Both datasets share some similarities in having short lines and many regressions. Nonetheless, the best performing algorithm was different in the two datasets, this is possibly because the pattern of regressions in reading source code is somewhat different from regressions in reading natural language text. Despite the fact that both EMIP and GazeBase had many between-line regressions, looking at the data it seems like natural language regressions are intended to re-read the text sequentially, where a jump back is followed by sequential reading. On the other hand, source code regressions are often followed by progressions or jumps forward instead of re-reading the code sequentially. Regress as an algorithm implicitly matches the pattern of sequential regressions that are observed in natural language reading, which might explain why it excels in correcting GazeBase data and under performs with source code. On the other hand, Attach is a simple algorithm that makes no assumptions on reading order, which might explain its success with correcting source code regressions. Nonetheless, the observation of different types of regressions motivates future research in this direction.

It is important to mention that the degree of non-linearity in reading source code is related to the level of programming experience as reported by Busjahn et al. (9). Therefore, we assume that there is a degree of sequential reading that is interrupted by regressions in reading source code, and this is the assumption that motivates this paper. The advantage of the hybrid Warp approaches is maximized when sequential reading is interrupted by regressions. Nonetheless, it is possible for some very non-linear trials to be corrected best by an algorithm that does not make the sequential reading assumption Warp makes. Algorithms like Attach might be more suitable for correcting trials with a large degree of non-linearity, but in this paper we focus on giving Warp the ability to handle regressions.

It is also important to mention that the EMIP and GazeBase datasets are somewhat different from typical eye movement datasets, as they contain many lines of text and somewhat small vertical spacing between lines. In reading experiments, it is common for large line spacing to be used to aid the process of drift correction. This decision might be possible for reading natural language text, but for source code eye tracking experiments aim for a natural presentation of the code in typical format such as a development environment. This adds to the complexity of correcting eye tracking data over source code.

Regarding one of our central questions: How often do real eye tracking trials contains regressions? And what type of regression is prevalent? Our results suggest that the majority of trials include at least one type of regression. In the datasets we used, the percentage of trials containing at least one type of regression was approximately 100%, with a prevalence for within-line regressions. This highlights the importance of handling regressions in automated correction algorithms, especially in tasks such as reading source code where 100% of trials contained within-line regressions and between-line regressions. Furthermore, the advantage of using hybrid algorithms was proportional to the presence of between-line regressions in the data. Therefore, we expect no advantage over Warp in correcting data without regressions.

In regard to runtime analysis, the number of fixations in the trial appears to be more influential on runtime than the type of distortion present. The runtime of hybrid(warp+attach) and hybrid(warp+chain) algorithms was faster than Warp, despite taking some steps to split regressions from fixations. The two algorithms gain an advantage when trials have regressions, as regressions are corrected with the faster Attach or Chain algorithms. Therefore, hybrid Attach and Chain algorithms have better runtime compared to Warp, and depending on the presence of between-line regressions they can offer better correction accuracy. At the same time, hybrid algorithms that use Regress or Stretch take significantly longer, since these algorithms have higher computational complexity.

### Recommendations

Based on the results of running our proposed algorithms and Warp on synthetic and real data, we make the following recommendations:

1. For correcting eye tracking data with source code and nonlinear reading, hybrid(warp+attach) was the best algorithm.
2. For automatically correcting eye tracking data with dense multi-line natural language text (such as GazeBase), hybrid(warp+regress) was the best algorithm.
3. When runtime is a priority, hybrid Attach or Chain algorithms offer a combination of high accuracy and fast runtime.
4. If 100% accuracy on the word level is required, manual human correction is the only way this level of accuracy can be achieved (with present correction algorithms).
5. Researchers can achieve better eye tracking accuracy by using a few lines of text stimuli with a large font and large line spacing, when possible.

### Conclusion

In this paper, we presented a family of correction algorithms for eye tracking data in reading source code that we generalize for some cases of natural language text as well. The proposed hybrid algorithms attempt to split regressions, correct the non-regressive fixations first, and then combine the corrected data with regressions to correct it with an algorithm that handles regressions well. We demonstrated that this hybrid approach matches or yields performance improvement over Warp in cases of synthetic and real data.

We make another contribution by presenting an external conceptual replication of Carr et al. (12), where our results match the original study and add to it. We continue the valued effort to make data and code available by making our code and data available through a replication package.

Future work is still needed to reach a higher accuracy in the automatic correction of eye tracking data especially with source code, and the hybrid ideas we present could be part of the incremental work towards the goal of matching the accuracy of experienced human correctors. Here we list some of the future directions that might improve the techniques presented:

1. For Hybrid algorithms, the regression detection idea presented appears to be sensitive to two types of distortions: noise and slope. Although Hybrid algorithms continue to outperform Warp, coming up with a more elaborate regression detection algorithm can improve the performance even further.
2. Correcting eye tracking data in reading source code appears to be a more challenging problem than reading natural language text due to the non-linearity. At the same time, source code has many characteristics that make algorithms specific for correcting eye tracking data in code reading viable. The variability in line length can aid the process of matching fixations to specific lines of code, for example.
3. Correction algorithms can take advantage of the different types of regression observed in real eye tracking data. In addition, future research could focus on identifying and comparing the different regression patterns and norms in reading source code and natural language text.
4. New and possibly better automated correction methods might be inspired from observing human correctors and the decisions they make in correcting eye tracking data.

### Ethics and Conflict of Interest

The author declares that the contents of the article are in agreement with the ethics described in http://biblio.unibe.ch/portale/elibrary/BOP/jemr/ethics.html and that there is no conflict of interest regarding the publication of this paper.

### Data Availability

Data and code including examples are available at the following OSF replication package:

https://osf.io/6s3vq/

### Acknowledgements

The author would like to thank undergraduate research students Brett Torra, Najam Tariq, Zaynab Tariq, Owen Raymond, Bishal Khadka, Nafis Bhuiyan, Yelaman Moldagali for their help in creating the golden set.
